# Supplementary material for: A fault-intrusion-tolerant system and deadline-aware algorithm for scheduling scientific workflow in the cloud
Source: PeerJ Comput Sci. 2021 Nov 2;7:e747. doi: 10.7717/peerj-cs.747 (PMC8576554; doi:10.7717/peerj-cs.747)
Supplement: Supplemental Information 6 [file peerj-cs-07-747-s006.zip › Myproject1.1/docs/org/cloudbus/cloudsim/provisioners/RamProvisionerSimple.html]

RamProvisionerSimple


JavaScript is disabled on your browser.


- Overview
- Package
- Class
- Use
- Tree
- Deprecated
- Index
- Help

- Prev Class
- Next Class

- Frames
- No Frames

- All Classes

- Summary:
- Nested |
- Field |
- Constr |
- Method

- Detail:
- Field |
- Constr |
- Method


org.cloudbus.cloudsim.provisioners

## Class RamProvisionerSimple

- java.lang.Object
- - org.cloudbus.cloudsim.provisioners.RamProvisioner
  - - org.cloudbus.cloudsim.provisioners.RamProvisionerSimple

- ---

    

  ```
  public class RamProvisionerSimple
  extends RamProvisioner
  ```

  RamProvisionerSimple is an extension of RamProvisioner which uses a best-effort policy to
  allocate memory to a VM.

  Since:
  :   CloudSim Toolkit 1.0

- - ### Constructor Summary

    Constructors

    Constructor and Description

    `RamProvisionerSimple(int availableRam)`

    Instantiates a new ram provisioner simple.
  - ### Method Summary

    Methods

    | Modifier and Type | Method and Description |
    | --- | --- |
    | `boolean` | `allocateRamForVm(Vm vm, int ram)` Allocates RAM for a given VM. |
    | `void` | `deallocateRamForAllVms()` Releases BW used by a all VMs. |
    | `void` | `deallocateRamForVm(Vm vm)` Releases BW used by a VM. |
    | `int` | `getAllocatedRamForVm(Vm vm)` Gets the allocated RAM for VM. |
    | `protected java.util.Map<java.lang.String,java.lang.Integer>` | `getRamTable()` Gets the ram table. |
    | `boolean` | `isSuitableForVm(Vm vm, int ram)` Checks if is suitable for vm. |
    | `protected void` | `setRamTable(java.util.Map<java.lang.String,java.lang.Integer> ramTable)` Sets the ram table. |

    - ### Methods inherited from class org.cloudbus.cloudsim.provisioners.RamProvisioner

      `getAvailableRam, getRam, getUsedRam, setAvailableRam, setRam`
    - ### Methods inherited from class java.lang.Object

      `clone, equals, finalize, getClass, hashCode, notify, notifyAll, toString, wait, wait, wait`

- - ### Constructor Detail


    - #### RamProvisionerSimple

      ```
      public RamProvisionerSimple(int availableRam)
      ```

      Instantiates a new ram provisioner simple.

      Parameters:
      :   `availableRam` - the available ram
  - ### Method Detail


    - #### allocateRamForVm

      ```
      public boolean allocateRamForVm(Vm vm,
                             int ram)
      ```

      **Description copied from class: `RamProvisioner`**

      Allocates RAM for a given VM.

      **Specified by:**
      :   `allocateRamForVm` in class `RamProvisioner`

      Parameters:
      :   `vm` - virtual machine for which the RAM are being allocated
      :   `ram` - the RAM

      Returns:
      :   $true if the RAM could be allocated; $false otherwise


    - #### getAllocatedRamForVm

      ```
      public int getAllocatedRamForVm(Vm vm)
      ```

      **Description copied from class: `RamProvisioner`**

      Gets the allocated RAM for VM.

      **Specified by:**
      :   `getAllocatedRamForVm` in class `RamProvisioner`

      Parameters:
      :   `vm` - the VM

      Returns:
      :   the allocated RAM for vm


    - #### deallocateRamForVm

      ```
      public void deallocateRamForVm(Vm vm)
      ```

      **Description copied from class: `RamProvisioner`**

      Releases BW used by a VM.

      **Specified by:**
      :   `deallocateRamForVm` in class `RamProvisioner`

      Parameters:
      :   `vm` - the vm


    - #### deallocateRamForAllVms

      ```
      public void deallocateRamForAllVms()
      ```

      **Description copied from class: `RamProvisioner`**

      Releases BW used by a all VMs.

      **Overrides:**
      :   `deallocateRamForAllVms` in class `RamProvisioner`


    - #### isSuitableForVm

      ```
      public boolean isSuitableForVm(Vm vm,
                            int ram)
      ```

      **Description copied from class: `RamProvisioner`**

      Checks if is suitable for vm.

      **Specified by:**
      :   `isSuitableForVm` in class `RamProvisioner`

      Parameters:
      :   `vm` - the vm
      :   `ram` - the ram

      Returns:
      :   true, if is suitable for vm


    - #### getRamTable

      ```
      protected java.util.Map<java.lang.String,java.lang.Integer> getRamTable()
      ```

      Gets the ram table.

      Returns:
      :   the ram table


    - #### setRamTable

      ```
      protected void setRamTable(java.util.Map<java.lang.String,java.lang.Integer> ramTable)
      ```

      Sets the ram table.

      Parameters:
      :   `ramTable` - the ram table


- Overview
- Package
- Class
- Use
- Tree
- Deprecated
- Index
- Help

- Prev Class
- Next Class

- Frames
- No Frames

- All Classes

- Summary:
- Nested |
- Field |
- Constr |
- Method

- Detail:
- Field |
- Constr |
- Method
